# Intraoperative Creation of Tissue-Engineered Grafts with Minimally Manipulated Cells: New Concept of Bone Tissue Engineering In Situ

**DOI:** 10.3390/bioengineering9110704

**Published:** 2022-11-17

**Authors:** Olga A. Krasilnikova, Denis S. Baranovskii, Anna O. Yakimova, Nadezhda Arguchinskaya, Anastas Kisel, Dmitry Sosin, Yana Sulina, Sergey A. Ivanov, Peter V. Shegay, Andrey D. Kaprin, Ilya D. Klabukov

**Affiliations:** 1 Department of Regenerative Medicine, National Medical Research Radiological Center, Koroleva St. 4, 249036 Obninsk, Russia; 2 Research and Educational Resource Center for Cellular Technologies, Peoples’ Friendship University of Russia (RUDN University), Miklukho-Maklay St. 6, 117198 Moscow, Russia; 3 Federal State Budgetary Institution “Centre for Strategic Planning and Management of Biomedical Health Risks” of the Federal Medical Biological Agency, Pogodinskaya St. 10 Bld. 1, 119121 Moscow, Russia; 4 Department of Obstetrics and Gynecology, Sechenov University, Bolshaya Pirogovskaya St. 2 Bld. 3, 119435 Moscow, Russia; 5 Obninsk Institute for Nuclear Power Engineering, National Research Nuclear University MEPhI, Studgorodok 1, 249039 Obninsk, Russia

**Keywords:** bone repair, cell therapy, in situ, minimally manipulated cells, stem cells, surgery, tissue engineering

## Abstract

Transfer of regenerative approaches into clinical practice is limited by strict legal regulation of in vitro expanded cells and risks associated with substantial manipulations. Isolation of cells for the enrichment of bone grafts directly in the Operating Room appears to be a promising solution for the translation of biomedical technologies into clinical practice. These intraoperative approaches could be generally characterized as a joint concept of tissue engineering in situ. Our review covers techniques of intraoperative cell isolation and seeding for the creation of tissue-engineered grafts in situ, that is, directly in the Operating Room. Up-to-date, the clinical use of tissue-engineered grafts created in vitro remains a highly inaccessible option. Fortunately, intraoperative tissue engineering in situ is already available for patients who need advanced treatment modalities.

## 1. Introduction

Translation of regenerative medical approaches into routine clinical practice is limited by strict legal regulations of cultured cells. Legal restrictions are intended to protect patients from the risks associated with substantial cell manipulation [1,2,3]. Therefore, tissue-engineered grafts created directly in the operating room (O.R.) with minimal manipulation of cells appear to be extremely promising. 

Intraoperative tissue engineering in situ demonstrated its effectiveness in recent clinical studies [4]. Traditionally, the term ‘tissue engineering in situ’ means different approaches to creating grafts that will mature into functionally active tissue inside the recipient’s body using its own regenerative potential [5]. However, we believe that the term ‘tissue engineering in situ’ should be applied to the process of intraoperative creation of tissue-engineered grafts with their implantation during the same surgical procedure. The obvious advantage of in situ tissue-engineered grafts is off-the-shelf availability, while traditional full-cycle tissue engineering requires sufficient time and financial expenses for cell culture in vitro. Importantly, tissue-engineered grafts created during surgical procedures can be enriched with cells isolated intraoperatively with minimal manipulation.

Traditional tissue engineering in situ does not include scaffold enrichment with cells since it requires in vitro culturing [5]. However, existing techniques and devices allow intraoperative cell isolation and cell seeding on scaffolds. The osteoconductive and osteoinductive properties of scaffolds are required for successful bone regeneration. However, the addition of a cellular component to various types of scaffolds can bring additional therapeutic value. Enrichment of scaffolds with intraoperatively isolated cells is an effective tool to create a unique milieu around the graft, enhance its maturation, and stimulate the intrinsic regenerative potential of the recipient’s body. In particular, seeding with bone marrow-derived mononuclear cells can contribute to angiogenesis in the implantation area [6]. Mesenchymal stem cells (MSCs) are one of the most important cell types within mononuclear and stromal vascular fractions. Tissue engineering in situ may benefit from MSCs due to their ability to reduce inflammation and promote angiogenesis. Also, scaffold enrichment with MSCs in situ may be of value due to the MSC potential for osteogenic differentiation. Calcium phosphate-containing scaffolds are shown to promote MSC differentiation into osteoblasts [7]. Notably, MSCs seeded on calcium phosphate-containing scaffolds underwent osteogenic differentiation and formed bone tissue in vivo earlier than embryonic stem cells [8]. Enrichment with stromal vascular fraction cells has also been shown to enhance the effectiveness of osteoinductive scaffolds in a critical-size cranial defect in mice [9].

**Figure 1:**
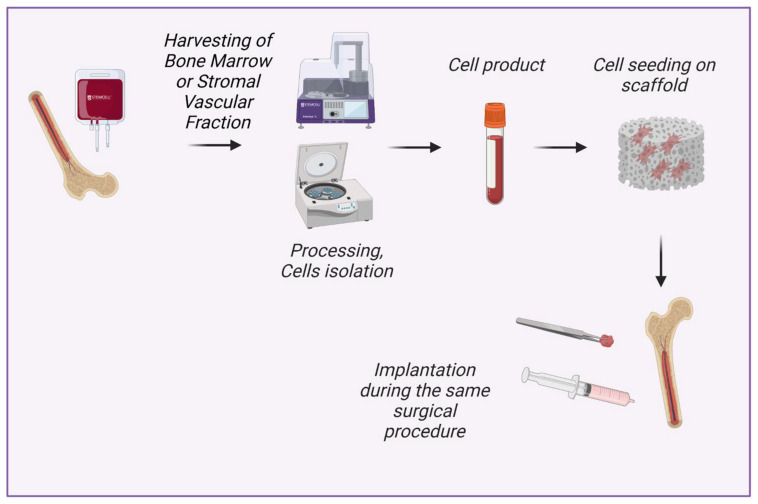
New concept of tissue engineering in situ: intraoperative isolation of cells with minimal manipulation, cell seeding on scaffold and implantation during the same surgical procedure. Created with BioRender.com.

Our understanding of tissue engineering in situ as a process of intraoperative creation of tissue-engineered graft comprises the possibility of scaffold enrichment with intraoperatively isolated minimally manipulated cells (Figure 1). These cells can be isolated intraoperatively from structural and non-structural tissues including bone marrow, adipose tissue, skin, mucous tissue, etc., seeded on the scaffold and implanted in a patient in a one-step procedure [10,11]. In contrast, tissue engineering with cultured cells requires two separate interventions (e.g., bone marrow aspiration/adipose tissue harvesting and surgical implantation of created tissue-engineered graft), authorized manufacturing facilities, and compliance with special regulations for advanced therapy medicinal products [2,12].

Our concept of intraoperative tissue engineering in situ does not contradict traditional tissue engineering in situ since the enrichment of scaffolds with intraoperatively isolated cells may contribute to the stimulation of the recipient’s own regenerative potential [13].

The aim of this review is to discuss the possible tools for intraoperative cell isolation and available approaches to fast-track scaffold enrichment as well as to conceptualize the use of the term ‘tissue engineering in situ’ for the description of intraoperative creation of tissue-engineered grafts.

## 2. Cells Used for Bone Tissue Engineering In Situ

The use of in vitro cultured cells has been shown to enhance bone healing in multiple studies. In particular, it helped to treat non-unions [14,15]. In this section, we will consider cells that have shown their effectiveness for bone regeneration and that can be isolated during a surgical procedure with minimal manipulations.

Cells contained in the bone marrow mononuclear fraction are often used to create tissue-engineered grafts in the operating room, although in some clinical studies scaffolds were enriched with bone marrow aspirate concentrate or with non-concentrated bone marrow aspirate [16,17].

Bone marrow mononuclear cells (BM-MNCs) are widely used in clinical practice alone or in combination with various scaffolds, including bone autografts, hydroxylapatite, β-tricalcium phosphate, etc. Multiple clinical and animal studies demonstrated results in improved bone healing [4,18,19]. Importantly, BM-MNCs are available as minimally-manipulated cells without preliminary cell culture [20]. BM-MNCs are a heterogeneous population of cells with a single nucleus, which includes mesenchymal stromal and stem cells, hematopoietic stem cells, endothelial progenitor cells, very small embryonic-like stem cells, embryonic stem cells, etc. The presence of different cell types in the mononuclear fraction causes combinative effects on regeneration. In particular, endothelial progenitor cells promote vascularization, and mesenchymal stem cells reduce inflammation.

Mesenchymal stem cells (MSCs) as well as mesenchymal stromal cells are components of BM-MNCs and one of the key players in tissue regeneration due to their ability to reduce inflammation, express growth factors, and differentiate into osteoblasts [21]. MSCs represent the population of osteoblasts progenitors in the bone marrow. Interestingly, ultrasound microwave irradiation was used for stimulation of MSCs osteo-differentiation in culture [22]. A combination of various scaffolds and cultured bone marrow-derived MSCs (BM-MSCs) has been used for the treatment of bone fractures or resorption in multiple clinical and animal studies [14,19,23,24]. Importantly, the combination of uncultured BM-MNCs and β-tricalcium phosphate resulted in enhanced bone regeneration and mineralization in vivo compared to MSCs cultured on the same scaffold [19].

For purposes of intraoperative tissue engineering in situ, MSCs can be isolated from other sources besides bone marrow. Intraoperative enrichment with adipose tissue-derived MSCs (AD-MSCs) could be achieved by the adhesion of stromal vascular fraction (SVF) cells to the scaffold. In that case, AD-MSCs will be delivered together with the other SVF components. The reparative success of SVF is also based on contained endothelial progenitor cells, M2-macrophages, smooth muscle cells, preadipocytes, etc. [25,26]. Those cells provide an essential microenvironment for MSCs and regulate their differentiation in an osteogenic direction via cell-to-cell cross-talks [27]. Also, known regenerative effects of SVF cells include improvement of angiogenesis and secretion of extracellular matrix. The chain of effects results in the ability of SVF to mineralize decellularized bone matrix and polycaprolactone and to increase new bone formation in vivo [9,28,29]. An experimental study in vitro showed that bone marrow-derived MSCs have even more osteogenic potential than adipose-derived cells [30]. However, multiple animal studies have shown no statistical difference between BM-MSCs and AD-MSCs in terms of bone regeneration [31,32]. At the same time, cell yield (incl. stem cell yield) is generally higher in adipose tissue than in bone marrow aspirate [33].

One of the promising directions is the isolation of MSCs from gingival tissues [34,35]. Some studies showed that gingiva-derived MSCs had better results in terms of staining for ALP; mineralized nodule formation; and APL, OSX, and RUNX2 gene expression compared to BM-MSCs in mice [36]. Among the advantages of isolating MSCs from the gingiva is minimal surgical intervention, as well as fast scarless healing of the gingival donor site [37]. Enzymatic treatment of gingival tissue takes around 2 h allowing the scaffold to be intraoperatively enriched with resulting suspension that contains gingiva-derived MSCs [37].

Sufficient vascularization is crucial for the regeneration of bone defects [38]. Endothelial progenitor cells (EPCs) have been shown to enhance vascularization in several studies [39,40]. EPC is included in the mononuclear fraction of bone marrow and also are found in SVF and peripheral blood.

## 3. Materials for Bone Tissue Engineering In Situ 

The type of implantable materials is a key factor in maintaining the shape and mechanical properties of the bone graft after implantation [41]. All materials for bone tissue engineering are mainly divided into non-resorbable (e.g., ceramic and polymeric grafts) and bioresorbable materials [42,43,44]. Various osteoinductive materials, such as beta-tricalcium phosphate (β-TCP) [45], hydroxyapatite [46], and xenografts [47] are commonly used as scaffolds in preclinical studies and clinical cases due to their commercial availability. Recent evidence also suggested bioactive glass ceramics as a suitable material for bone tissue engineering, it being capable of the promotion of osteogenesis [48].

Notably, hydroxyapatite can also be in a resorbable form. The effectiveness of resorbable high-porous acellular hydroxyapatite scaffolds is generally based on the enhanced local regenerative potential [49,50]. Resorbable scaffolds could be a suitable solution for cartilage and bone tissue engineering [51,52,53]. While the mobilization of patients’ own osteoblasts leads to the revitalization of the graft, the scaffold could be completely replaced by a newly formed extracellular matrix.

**Figure 2:**
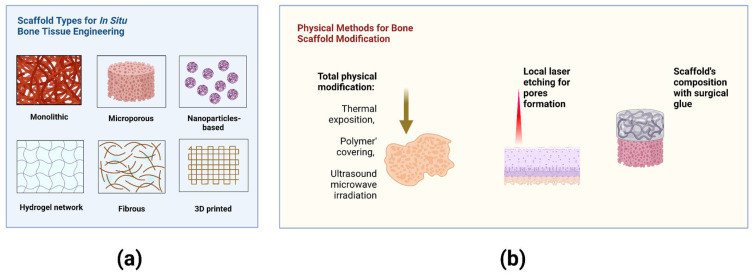
(**a**) Scaffold types for bone tissue engineering in situ. (**b**) Methods for physical modification of scaffolds immediately in the Operating Room. Created with BioRender.com.

Despite numerous types of scaffolds, bone graft autotransplantation is still considered a ‘gold-standard’ approach to repairing bone defects (Figure 2a). However, a well-known method of autologous osteoplasty is commonly limited by the harvested bone volume and associated with an additional risk of complications in the donor site [20,54].

The material-related complications remain to pose a sufficient risk due to individual response after implantation [55,56]. Insufficient vascularization and inflammatory response are often observed in the bone regeneration zone [57]. Bone tissue engineering in situ through BM-MSCs osteogenic differentiation may be performed using immobilized gene delivery nanocomplexes [58]. Bioactive scaffolds with modified surface properties or gene-activated materials have promising applications as scaffolds for cell seeding [59,60].

Promising physical approaches allow the modification of biomaterials and cells immediately in the Operating Room (O.R.). Laser engraving of cartilage led to improved deep chondrocyte migration into the scaffold after implantation [52,61]. The nonselective damaged cartilage also stimulated cell migration via cell secretion activity [62,63]. The methods for modification of bone scaffolds and their properties are summarized in Figure 2b.

Various materials are used in bone tissue engineering, and, notably, the adhesion capacity of BM-MNCs differs depending on the type of material. Henrich et al. (2015) showed that coating of β-TCP with fibronectin and human plasma does not increase the adhesion capacity of human BM-MNCs isolated by density gradient centrifugation, at the same time the percentage of attached cells was higher in β-TCP and demineralized bone matrix compared to bovine cancellous bone [64]. The comparative effectiveness of implantation of various materials for the treatment of large bone defects in rats showed that the fibrous demineralized bone matrix seeded with centrifugation-isolated BM-MNCs led to promising results in comparison with syngeneic cancellous bone implantation [65]. Centrifugation-isolated BM-MNCs were seeded on human demineralized bone matrix, bovine cancellous bone hydroxyapatite ceramic, and β-tricalcium phosphate scaffolds. The study showed that the effectiveness of BMC-supported therapy could be influenced by the type of scaffold. Although the demineralized bone matrix was superior in comparison to β-tricalcium phosphate and bovine cancellous bone hydroxyapatite ceramic, the level of autologous bone could not be attained [66].

## 4. Methods of Intraoperative Cell Isolation

Various cell isolation/separation methods have been developed to date. However, minimal requirements for intraoperative application usually combine fast-track processing of large tissue volume with the high viability of isolated cells. Also, the method has to be in step with actual regulations for clinical use. Next, we will focus on cell isolation methods that can be used in the operating room. Importantly, the regulations regarding acceptable methods of intraoperative cell isolation and clinical use of obtained cells may be different in various countries. Most commonly, cells can be intraoperatively obtained using density gradient centrifugation and selective cell retention. Multiple time- and effort-saving devices for automatic cell processing are becoming common in clinical practice.

In some studies, non-concentrated bone marrow aspirate was applied to the scaffold for enrichment with BM-MNCs [17,67].

However, in some cases, bone marrow aspirate is processed for the concentration and isolation of BM-MNCs. The Ficoll density gradient centrifugation and its modified versions are among the widespread and simple techniques for BM-MNC concentration [68]. To date, various concentration devices have been created for automatic cell processing. Most time-efficient examples were reported to concentrate BM-MNCs in 90 s [69]. Sepax is one of the most commonly used closed automatic BM-MNC isolation devices. Sepax can also be used for the automatic isolation of SVF cells allowing higher cell yield compared to the manual isolation method [70].

Cells can be isolated and seeded into various scaffolds using selective retention technologies [71]. Selective cell retention (SCR) is used to concentrate osteogenic progenitor cells on the scaffold and is based on the adhesive properties of the scaffold surface and size-dependent pump-assisted filtering of cells through scaffold volume. Henze et al. (2019) demonstrated that the surgical suction filter can accumulate MSCs during joint replacement surgery [72]. In the study by Busch et al. (2020) porous bone substitute material was placed in the suction device, and the accumulation of mononuclear cells (MNCs), including MSCs, epidermal growth factor, platelet-derived growth factor, and other cytokines in bone substitute material was confirmed [73].

The Screen–Enrich–Combine(-Biomaterials) Circulating System also allows intraoperative enrichment of scaffold with autologous MSCs in 10–15 min. The device is based on the circulation of bone marrow through a porous β-TCP scaffold that acts as a filter to which MSCs adhere [74]. Wang et al. showed that besides MSCs platelets also adhered to porous β-TCP using Screen–Enrich–Combine Circulating System [75]. Jacobsen et al. also demonstrated the use of a CellectTM cell retention device that allowed the enrichment of the matrix for bone repair with progenitor cells from bone marrow aspirate [76]. Importantly, a comparative study of density separation and selective retention techniques revealed that both processing methods helped to achieve successful bone regeneration [77]. Sometimes, the technology of selective cell retention is enhanced by the incorporation of specific ligands for cell adhesion in the scaffold. In the study by Luo et al., the demineralized bone matrix was modified with integrin ligands that have an affinity to integrins of BM-MSCs [78].

The method of negative adhesion-based selection requires the filter coating with special ligands that have an affinity to non-target cells eliminating them from the heterogeneous cell suspension. As a result, only targeted cells remain in the final suspension. To achieve such negative selection the affinity of ligands to different types of cells in the suspension must be known. 

Stromal vascular fraction (SVF) cells can be isolated from lipoaspirate by enzymatic treatment, non-enzymatic treatment with centrifugation or inter-syringe dissociation, and using automated devices for point-of-care isolation [70,79,80,81]. Doi et al. (2013) described an automatic system for SVF cell suspension preparation in 65 min. Notably, cell yield between the manual separation group and the automatic separation group did not differ [80]. However, the use of the Sepax device allowed the isolation of a larger number of SVF cells than the manual method [70].

Stromal vascular fraction components can be further separated using the dielectrophoresis approach [82]. Dielectrophoresis (DEP) is a flow-based method for cell separation. It is based on dielectrophoretic force application that is perpendicular to the flow axis separating various cell types on sufficient differences in the density and dielectric characteristics [83]. In the study by Vykoukal et al., a dielectrophoretic field-flow fractionation device was used to isolate progenitor cells from debris, damaged cells, erythrocytes, and other components of SVF in approximately 20 min [82]. However, the use of dielectrophoresis for cell isolation in an intraoperative setting requires further modification of technology to allow the processing of large volumes of tissue.

Methods that can be potentially used for intraoperative cell isolation are summarized in Table 1.

## 5. Methods of Intraoperative Cell Seeding on Scaffolds 

The time for cell isolation and seeding is limited by surgical procedures. One of the important questions is what time is sufficient for effective cell adhesion to the scaffold. If adhesion is achieved, the odds of cell persistence in the transplantation area are increased. Previously, we showed that 20 min incubation in cell suspension was enough for the adhesion of BM-MNCs on bone material [20]. In another study, fluorescence microscopy confirmed that rat BM-MNCs adhered to different types of scaffolds after 10 min of incubation at 37 °C [65].

One of the most commonly used intraoperative cell seeding methods is a static incubation of scaffold in a cell suspension. In some studies, bone marrow aspirate concentrate or bone marrow aspirate were placed on the scaffold for 15–20 min for cell adhesion and further implantation of the cell-enriched construct [16,67]. Dynamic cell seeding methods have been developed to enhance cell adhesion. In particular, the use of centrifugal force helped to achieve high colonization with HT-29 cells in 5 min. The method implies that under the centrifugal force cells are pushed into the volume of the scaffold [87]. However, we have not found animal or clinical studies in which dynamic cell seeding was used for creation of tissue-engineered grafts in situ. The question about optimal time and speed needs to be further investigated since no studies used minimally manipulated cells.

To improve the colonization of the scaffold, cells were injected into the volume of the scaffold using a needle [88,89] or were seeded on the scaffold in combination with fibrin [9,90,91]. However, some studies showed that coating of β-TCP with fibronectin and human plasma does not increase the adhesion capacity of human BM-MNCs [64].

Method of selective retention/filtration combines cell isolation and cell seeding and allows scaffold enrichment in 10–15 min. The method is based on the circulation of cells through a scaffold that serves as a filter for cell adhesion. The selective cell retention technique can lead to enhanced cell adhesion to the scaffold (filter) if the scaffold is enriched with cell-specific ligands. The retention/filtration approach may be realized in the form of a newly developed perfusion bioreactor, in which the scaffold is placed in the perfusion chamber through which the cells go with the medium flow and adhere. Notably, various seeding modes are available [92]. The retention/filtration method has been described above in more detail.

3D printing is a promising technique for the formation of personalized grafts for cartilage and bone regeneration using hydrogels, bone chips, laser-sintered titanium [93,94,95,96]. Traditionally, 3D bioprinting involves cells cultured in vitro. However, Gettler et al., showed that SVF cells can be successfully incorporated into the collagen and used as bioink for 3D bioprinting [97]. As mentioned above, SVF can be isolated intraoperatively, opening new ways for its use in intraoperative 3D bioprinting. Potentially, bone marrow aspirate cells or bone marrow mononuclear cells isolated intraoperatively may also be incorporated into the bioink for intraoperative production of 3D bioprinted grafts. Several successful attempts have been made to adapt various bioprinting technologies for intraoperative tissue engineering [98,99]. Laser-assisted bioprinting with photoactivated gel could become a reliable tool in orthopedic surgery in the future for osteochondral repairing and cartilage engineering in situ [100,101]. However, existing examples of its application for bone engineering in situ are extremely rare. Intraoperative 3D-bioprinting in situ can be performed using cell-injectable pens [102]. Handheld bioprinting could help clinicians to fill the small volume gap in a patient’s bone with bioink, while the majority of bone lesions require replacement with the graft and exceed the limits of bioprinting in situ [103].

Cell adhesion may also be enhanced via modification of scaffolds, for example, scaffold immersion in deionized water [104]. Modification of the material’s surface by integrin-binding ligands and laser engraving of surfaces also led to improved cell adhesion [52,105].

Various cell isolation and seeding methods have been developed to date. At the same time, an important aspect is the safety of using a particular method in clinical practice. Questions about the possible impact of certain methods on the cell’s therapeutic properties and safety need further thorough investigation.

Methods of cell seeding that can be potentially used for intraoperative scaffold enrichment are presented in Table 2.

## 6. Clinical Application

In various clinical studies, tissue-engineered grafts have been created directly during the surgical procedure. In the study by Ahn et al., (2018) 3D printed personalized scaffold was incubated for 20 min with not concentrated bone marrow aspirate isolated intraoperatively during the procedure of cleft alveolus reconstruction [67]. Implantation of 3D scaffold enriched in bone marrow cells resulted in the formation of new bone in 45% of total defect volume 6 months after surgery.

More often, bone marrow aspirate undergoes concentration to improve therapeutic cells concentration and eliminate unwanted components. Bone marrow aspirate concentrate (BMAC) contains mesenchymal stromal and stem cells, endothelial progenitor cells, hematopoietic stem cells, platelets, cytokines, and growth factors such as platelet-derived growth factor, vascular endothelial growth factor, bone morphogenetic proteins [107,108]. A total of 39 patients with bone defects received either collagen or hydroxyapatite scaffold enriched in BMAC in a prospective clinical trial by Jäger et al. It has been shown that complete bone healing was achieved earlier in the HA group compared to collagen [16]. Hendrich et al. (2009) also showed that intraoperatively isolated BMAC can be cultivated with calcium phosphate scaffolds before implantation. Importantly, it has been shown that scaffold enrichment with BMAC cells does not cause complications such as infections, excessive new bone formation, and malignization in patients [109]. BMAC was obtained after 15 min of bone marrow aspirate centrifugation and intraoperatively seeded on the bovine xenogenous scaffold in the study by Petri et al. (2013) Implantation of an in situ created tissue-engineered construct led to the healing of segmental long-bone defects >3 cm in 4 out of 5 patients [110]. Importantly, similar overall rates of nonunions healing were noted between cancellous allograft enriched with BMAC and gold-standard iliac crest bone graft in the study by Lin et al. (2019) [111].

Vadala et al. (2008) reported the intraoperative creation of tissue-engineered grafts for implantation in elderly osteoporotic patients. Enrichment of corticocancellous bone allograft with platelet-rich fibrin and BM-MNCs isolated intraoperatively using a two-step centrifugation approach helped to achieve cervical fusion 6 months after the procedure [112]. Intraoperative enrichment of β-TCP with BM-MNCs isolated intraoperatively in 15 min of centrifugation was used to repair the alveolar cleft in the study by Du et al., (2017). Authors reported a reduction in postoperative pain and hospital stay days [4]. Al-Ahmady et al. (2018) demonstrated that a higher percentage of alveolar bone unions was achieved using an intraoperatively created graft consisting of centrifugation-isolated BM-MNCs seeded on a collagen-nanohydroxyapatite-platelet-rich fibrin scaffold compared to grafting with iliac crest bone [89].

Works comparing various techniques for cell isolation and scaffold enrichment are of particular value. Concerns regarding the insufficient number of therapeutically active cells in uncultured bone marrow remain one of the main factors causing skepticism towards cells that were not in vitro expanded. Du et al. (2021) conducted important research showing that concentrated BM-MNCs seeded on β-TCP scaffold led to more bone regeneration in Beagles in comparison with in vitro expanded BM-MSCs seeded on the same scaffold [19].

Tissue-engineered grafts intraoperatively obtained by cell seeding on the scaffold with the use of selective retention/filtration approach not only were tested in animal studies [113] but also were used for bone repair in patients with various bone pathologies. Implantation of β-TCP intraoperatively seeded with MSCs using Screen–Enrich–Combine Circulating System led to a higher percentage of newly formed bone in the critical size defect of the goat tibia [114] and in patients with fractures with depressed tibial plateau compared to β-TCP alone [11]. The effectiveness of diaphyseal bone non-unions treatment with β-TCP scaffold intraoperatively enriched using Screen–Enrich–Combine Circulating System in 10 min was comparable to bone autograft 9 months after the procedure. Importantly, the use of a cell-enriched β-TCP scaffold helped to avoid donor site morbidity [75]. A retrospective study by Yang et al. (2021) showed that manual selective cell retention technology allowed intraoperative seeding of allogeneic bone material with bone marrow cells in 20 min. Created tissue-engineered constructs were used in spinal fusion in adolescents with idiopathic scoliosis and showed its safety and effectiveness [106]. Jacobsen et al. (2008) presented the case of ankle fusion in a patient with talus necrosis performed using a DBM graft that was intraoperatively enriched in bone marrow cells with selective retention device [76].

Vascularization of implanted bone grafts is essential for a successful graft take-up and function. Vasculogenic bone implants were created in 3 h by enrichment of the β-TCP or hydroxyapatite scaffold with freshly isolated stromal vascular fraction (SVF) cells and fibrin. Implantation in the mouse model led to the formation of blood vessels connected to the host’s vasculature, however, no frank bone was observed presumably due to the absence of SVF cell commitment to the bone lineage [115]. The addition of bone morphogenetic protein 2 (BMP-2) to SVF cells in intraoperatively created bone tissue-engineered graft promoted the differentiation of progenitor cells into osteoblasts that leads to the formation of bone tissue in mice [116]. In the study by Nyberg et al. (2019) intraoperatively created SVF-containing scaffolds helped to achieve improved bone healing in vivo compared to acellular scaffolds [9]. The technology of intraoperative scaffold enrichment with SVF cells has been used to treat fractures in humans. Intraoperatively created tissue-engineered constructs consisting of ceramic granules and SVF cells were used to treat fractures of the proximal humerus [117].

## 7. Discussion

In this review, we expanded the common understanding of tissue engineering in situ. Traditionally, this concept was used in a limited paradigm of scaffolds and bioactive molecules allowing subsequent endogenous cell recruitment and tissue maturation in the recipient’s body. However, we have shown the novel possibilities of intraoperative cell isolation and seeding on various scaffolds for the immediate creation of tissue-engineered graft and its implantation. We suppose that the intraoperative creation of tissue-engineered grafts can be reasonably named tissue engineering in situ.

The maturation of intraoperatively created constructs also takes place in the host’s body. At the same time, in-situ-created grafts are designed to enhance a patient’s regenerative potential. For example, enrichment of bone grafts with a cellular component can promote angiogenesis [6], therefore, uncultured cells are promising candidates for modification of scaffolds for tissue engineering in situ. Homing towards the injury site is one of the essential properties of various cell types, including MSCs [118]. Intraoperatively isolated cells maintain their properties and are capable of responding to chemoattractants. This ability may possibly reduce the risk of cell dissipation from the graft implantation area.

Autologous bone grafting remains a gold standard for the treatment of bone defects [119,120]. Isolation and use of bone fragments are performed in a one-step surgical procedure and do not require methods for material modification or processing. However, autologous bone grafting is a traumatic procedure and it cannot be used for the treatment of large bone defects [121]. Implantation of in-situ-created tissue-engineered graft represents a promising approach for bone regeneration, and it can help to avoid donor site morbidity that occurs during autologous bone grafting. The technology of intraoperative cell isolation with minimal manipulations has been developed long ago. Currently, there is a revival of interest in the in situ creation of tissue-engineered bone grafts. Tissue engineering in situ with the use of minimally manipulated cells seems a safer approach than with the use of in vitro cultured cells since cells do not undergo substantial manipulations that can have an impact on cells. Interestingly, enzymatic digestion is considered minimal manipulation in Russia and may represent more than minimal manipulation in USA regulation [10]. The success of tissue engineering in situ largely depends on the speed of cell isolation, cell number, and viability, and the speed and efficiency of adhesion of the cells to the scaffold. Further development of tissue engineering in situ requires continuous improvement of technologies for cell isolation and scaffold enrichment. New cell separation methods such as microfluidic inertial-ferro-hydrodynamic cell separation and density resolution of cells in spiral microfluidic channels have been developed [122,123,124]. However, cell separation in microfluidic channels requires prolonged separation time. Therefore, its use in intraoperative settings for clinical application is limited. Perhaps, with further development of technology, such methods can be used for intraoperative cell isolation. To be used for tissue engineering in situ, the developed methods should ensure the isolation of a sufficient number of cells in a short time, and also not adversely affect the properties of the cells for safety reasons [125]. Extremely promising methods for enhancement of cell adhesion on scaffolds are presented by a surface micro/nano-imprinted patternization and laser engraving of surfaces of the implant [52,126]. It can also be hypothesized that intraoperative 3D bioprinting with the use of minimally manipulated cells will allow fast and financially affordable creation of personalized tissue-engineered constructs.

The use of minimally manipulated cells for tissue engineering in situ is a promising approach. However, bone regeneration can also be promoted by bone morphogenetic proteins (BMPs), transforming growth factor (TGF-β), vascular endothelial growth factor (VEGF), fibroblast growth factor 2, insulin-like growth factor I [127,128,129,130]. Recombinant human BMP-2 and BMP-6 are used for the functionalization of materials for bone repair [131,132]. Transforming growth factor (TGF-β) controls bone formation by increasing proliferation and osteoblastic differentiation, and is also used for bone scaffolds enrichment [133,134]. It has been shown that injection of bone morphogenetic protein 2 (BMP-2) stimulates the differentiation of mesenchymal stromal cells and migration of osteoblasts [135]. Sufficient angiogenesis is important for successful bone healing. Modification of scaffolds with VEGF is a promising method that contributes to the blood supply and avoidance of delayed bone healing [128]. The use of fibroblast growth factor 2 and insulin-like growth factor 1 also contributed to bone healing in animal models [129,130]. Possibly, combining minimally manipulated cells with scaffolds enriched in morphogenetic proteins or growth factors can further enhance the effectiveness of tissue engineering in situ.

Another method for the enhancement of bone regeneration is the use of platelets. Platelets are known for containing a cocktail of therapeutic molecules in their granules, such as fibronectin, platelet-derived growth factor, vascular endothelial growth factor, transforming growth factor, epidermal growth factor, insulin-like growth factor, etc. [136]. Notably, platelet-rich plasma has been shown to enhance bone healing [137]. Typically used platelet preparations include platelet-rich plasma (PRP) and platelet-rich fibrin [138], which can also be prepared intraoperatively and potentially used for the enrichment of scaffold for bone repair. Importantly, platelets can promote the therapeutic potential of MSCs by enhancing MSC angiogenic function [139].

One of the concerns regarding tissue engineering in situ is a lower number of intraoperatively isolated minimally manipulated cells compared to in vitro expanded cells. One of the solutions to this problem is the development of non-traumatic methods of isolation that will reduce cellular stress and improve the effectiveness of constructs seeded with low doses of cells. At the same time, some studies show that there are no strong correlations between the amount of administered cells and the therapeutic effect [140]. In fact, recently, in vitro cultured cells exerted promising therapeutic results when administered at low doses of less than 40 × 10^3^ cells per 1 cm^2^ of a skin wound surface [141]. Interestingly, in a rat critical-sized bone defect, seeding of β-TCP with minimally manipulated BM-MNCs increased the volume of a newly formed bone, however, seeding of 5 × 10^6^ cells led to more pronounced bone formation compared to 1 × 10^7^ cells, and the highest mineral bone density was observed in β-TCP seeded with 1 × 10^6^ cells [140]. In some cases, bone defects may display delayed healing dynamics. Interestingly, Woloszyk et al. (2022) showed that the outcome of fracture healing was determined already at the hematoma stage [142]. Although currently there is no clear answer to the question of the optimal number of cells required to achieve better regenerative outcomes, recent studies not only confirm the benefits of tissue-engineered constructs created in situ but also suggest that low-dose cell therapy may also be effective.

In general, the current number of papers on intraoperative or tissue engineering in situ is limited; it is suggested that the efficiency of tissue-engineered constructs created in vitro is higher compared to those created in situ. However, the efficiency of tissue-engineered constructs created in situ may be improved by using more advanced isolation and seeding techniques. Routine clinical use of tissue-engineered constructs created in vitro remains an extremely inaccessible option due to strict regulations and high costs. At the same time, in situ tissue-engineered constructs are available for patients who need advanced treatment modalities immediately.

## 8. Limitations

The methods used for cell isolation are limited by the endogenous cell stress resistance needed for the maintenance of cell viability, secretion, and general homeostasis. Centrifugation is one of the most commonly used methods of cell isolation. By taking a typical cell density as 1.078 g/cm^3^ and a character cellular high as 10 μm (the nucleus size), we calculated that centrifugation at 14,000× *g* in saline is equivalent to the 100 Pa of additional pressure, meanwhile, a stress of 100 Pa is sufficient to produce a 10% strain [143]. A decrease in cellular stress may be achieved by using buffer solutions of density higher than that of saline, however fine adjustment of density is required. However, accelerations above 3000 g and time periods longer than 30 min are not usually required which allows to avoid cell damage. Under some circumstances, even with minimal manipulation cells are subjected to a certain stress during isolation. In particular, the use of enzymes for cell isolation can affect the expression of cell-surface molecules [144]. Hyperthermia is used to increase enzymatic processing efficiency and may be applied as a method of cellular isolation, however, it leads to cellular stress [145,146].

The issue of cell stress during isolation is closely related to the problem of the number of isolated cells. Despite the presence of studies showing that the greater number of isolated cells does not always correlate with better outcomes [140], the number of cells isolated in an intraoperative setting still causes concerns. One of the promising directions is to find methods to reduce cell damage during intraoperative isolation and to increase the number of isolated viable and functionally active cells.

To date, various promising methods of cell separation have been developed, however, some technologies do not allow fast isolation of clinically relevant cell numbers. The development of advanced techniques adapted for intraoperative use is crucial.

The use of particular intraoperative techniques of cell isolation and seeding may be limited by legal regulations that may be different in various countries. Additional studies of various methods of cell isolation, seeding and, possibly, stimulation, are needed to determine if particular methods may influence cell safety and quality. If studies confirm the absence of impact on cell safety and quality, the list of methods that are considered minimally manipulative may possibly be expanded.

## 9. Future Directions

1. Bone marrow and adipose tissue are the most common sources of intraoperative cell isolation. At the same time, there are other promising sources of mesenchymal stem and stromal cells, for example, gingival tissue. More active use of intraoperatively isolated gingiva-tissue-derived cells may become a promising direction in tissue engineering in situ.
2. Development of new methods and devices that will allow the gentle isolation of a greater number of viable cells. It has been shown that MSCs can endure exposure to hypotonic conditions (>30 min) without loss of cell potential and with only reversible deterioration in cell proliferation [147]. The method titled ‘osmotic selection’ can be further studied to investigate possibilities of intraoperative MSCs isolation based on MSCs resistance to osmotic lysis.
3. Currently available techniques of cell isolation, such as dielectrophoretic isolation, may be further modified to allow fast intraoperative processing of large volumes of tissue.
4. It can be hypothesized that intraoperative stimulation of the cells may affect further cell destiny. For example, platelets have been shown to stimulate the regenerative potential of MSCs, thus enriching scaffolds with two cell types or safe cell stimulation may possibly have additional value.

## 10. Conclusions

We proposed to use the term ‘tissue engineering in situ’ for the description of the process of intraoperative creation of tissue-engineered constructs with the use of minimally manipulated cells. Current methods of intraoperative cell isolation and seeding allow in situ creation of effective tissue-engineered constructs for bone regeneration. The use of intraoperatively isolated minimally manipulated cells not only creates a unique therapeutic milieu around implantable scaffolds but also is not subjected to strict legal regulations applied to cultured cells. Further progress in tissue engineering in situ requires the development of new techniques to achieve high cell yield and viability during intraoperative isolation and seeding.

## Figures and Tables

**Table 1:** Methods for intraoperative cell isolation for in situ tissue engineering.

No	Method	Principle	Examples of Devices and Systems	Refs.
1	Centrifugation	Separation based on differences in cell density by centrifugation.	Sepax, Percoll, Ficoll, etc.	[84,85]
2	Selective Retention/Filtration	Cell filtration through scaffold using adhesive properties of scaffold surface and size-dependent pump-assisted filtering of cells.	Screen-Enrich-Combine(-Biomaterials) circulating system, Cellect^TM^ cell retention device.	[74,76]
3	Adhesion-based negative selection	Adhesion-based depletion of non-target cells for enrichment of suspension with target cells.	-	[86]
4	Selective retention with the use of binding ligands	Addition of ligands to the scaffold to enhance cell adhesion.	-	[78]
6	Dielectrophoresis	Dielectric force separates cells depending on density and dielectric characteristics	-	[82]

**Table 2:** Methods that can be applied for intraoperative cell seeding.

No	Method	Principle	Time to Cell Adhesion	Refs.
1	Static cell seeding	Cell precipitation in suspension.	from 15–20 min	[16,67]
2	Dynamic cell seeding	Centrifugal force pushes cells into the volume of the scaffold	Approx. 5 min	[87]
3	Selective retention/filtration	Cell perfusion through porous scaffolds and adhesion	10–20 min	[74,106]
4	3D bioprinting	Cell incorporation into bioinks for 3D bioprinting.	30+ min	[97]
5	Cell injection into the scaffold center with the needle	Cells are injected into the inner volume of a scaffold to prevent cell loss.	Approx. 5 min	[88,89]
7	Cell combination with fibrin	Cell incorporation in fibrin prevents cell diffusion into adjacent tissues.	Approx. 5 min	[9,90]

## Data Availability

Not applicable.
